# Inhibition of Multidrug Efflux Pumps Belonging to the Major Facilitator Superfamily in Bacterial Pathogens

**DOI:** 10.3390/biomedicines11051448

**Published:** 2023-05-15

**Authors:** Manuel F. Varela, Jerusha Stephen, Deeksha Bharti, Manjusha Lekshmi, Sanath Kumar

**Affiliations:** 1 Department of Biology, Eastern New Mexico University, Station 33, Portales, NM 88130, USA; 2 ICAR-Central Institute of Fisheries Education (CIFE), Mumbai 400061, India; jeruselva@gmail.com (J.S.); dxa.bharti201713@gmail.com (D.B.); manjusha@cife.edu.in (M.L.); sanathkumar@cife.edu.in (S.K.)

**Keywords:** multidrug efflux pumps, antimicrobial transporters, major facilitator superfamily, bacterial pathogens, multidrug resistance, bacterial resistance, drug resistance

## Abstract

Bacterial pathogens resistant to multiple structurally distinct antimicrobial agents are causative agents of infectious disease, and they thus constitute a serious concern for public health. Of the various bacterial mechanisms for antimicrobial resistance, active efflux is a well-known system that extrudes clinically relevant antimicrobial agents, rendering specific pathogens recalcitrant to the growth-inhibitory effects of multiple drugs. In particular, multidrug efflux pump members of the major facilitator superfamily constitute central resistance systems in bacterial pathogens. This review article addresses the recent efforts to modulate these antimicrobial efflux transporters from a molecular perspective. Such investigations can potentially restore the clinical efficacy of infectious disease chemotherapy.

## 1. Bacterial Pathogens

Bacterial pathogens of concern within the context of the worldwide emergence and spread of antimicrobial resistance (AMR) are classified under the group ESKAPEE, comprising *Enterococcus faecium*, *Staphylococcus aureus*, *Acinetobacter baumannii*, *Klebsiella pneumoniae*, *Pseudomonas aeruginosa*, *Enterobacter* species, and *Escherichia coli* [1]. These bacterial species represent some of the extremely drug-resistant strains that threaten the relevance of existing antimicrobial therapy due to their current ability to resist all available antimicrobials [2]. ESKAPEE pathogens are the major cause of healthcare-associated infections (nosocomial), and it is estimated that more than 80% of global deaths are due to ESKAPEE pathogens [3].

The Gram-positive enteric bacterium *Enterococcus faecium* is the causative agent of neonatal meningitis, endocarditis, and bacteremia and is a common agent of nosocomial infections, second only to staphylococci [4]. The bacterium is intrinsically resistant to many antibiotics, such as the β-lactams and the cephalosporins, due to the overproduction of low-affinity penicillin-binding proteins (PBPs), aminoglycosides, and trimethoprim-sulfamethoxazole. In contrast, the bacterium has acquired resistance to several others, such as vancomycin, linezolid, tigecycline, and daptomycin, through point mutations and the acquisition of resistance plasmids [5,6]. Hospital environments, including medical devices, offer ideal niches for colonization by vancomycin-resistant enterococci (VRE), making these common sources of potentially fatal nosocomial infections in susceptible populations [6,7]. Enterococci are among the high-priority pathogens owing to the rapid increase in infections attributed to drug-resistant isolates [8].

*Staphylococcus aureus* is the most common human and animal pathogen responsible for skin, soft tissue, wound infections, life-threatening pneumonia, endocarditis, and infections associated with indwelling devices in hospital settings [9,10]. This bacterium is of great importance due to its resistance against numerous antimicrobial agents, and *S. aureus* has been involved in more than 100,000 deaths attributable to methicillin-resistant *S. aureus* (MRSA AMR in 2019 [3]. Although MRSA microorganisms were initially identified as healthcare-associated (HA-MRSA) strains, subsequently, distinct lineages of MRSA emerged from livestock and the community, termed livestock-associated (LA-MRSA) and community-associated (CA-MRSA) [11]. Due to the high genetic and phenotypic variation and their ability to adapt to various environmental conditions, staphylococci have become resistant to the most currently used antimicrobials. MRSA strains are typically resistant to β-lactams and cephalosporins, making it necessary to use alternate antibiotics such as glycopeptides (vancomycin, teicoplanin), tigecycline, daptomycin, and linezolid [12,13]. With the emergence of clonal types of MRSA resistant to each of these antimicrobials, the treatment options against this pathogen are becoming critically constricted to a minority of individual antibiotics or combination therapies involving more than one antibiotic [14].

The Gram-negative pathogens *Klebsiella pneumoniae, Acinetobacter baumannii, Pseudomonas aeruginosa,* and *Enterobacter* spp. (*Enterobacter cloacae* complex, ECC) are high-priority pathogens owing to their plethora of mechanisms to resist multiple antibiotics, colonize hospital environments and devices, and cause serious nosocomial infections [1]. *K. pneumoniae* ranked third in global deaths associated with AMR in 2019 [3]. Resistance to all β-lactam antibiotics, including cephalosporins, carbapenems, aminoglycosides, and fluoroquinolones, has left clinicians with few options to treat infections by these pathogens [4,15,16]. The carbapenem-resistant Enterobacterales (CRE) group, producing KPC, VIM, IMP, and NDM carbapenemases, is often associated with severe infections with a fatality rate as high as 40% [17]. Similarly, *P. aeruginosa* is a serious pathogen causing various infections, from wound infections to fatal bacteremia, pneumonia, and lung infections in patients with cystic fibrosis and in immunocompromised individuals. This bacterium possesses diverse drug-resistance mechanisms, both intrinsic and acquired, and produces antibiotic degradative enzymes such as AmpC lactamases, extended spectrum-β-lactamases (ESBLs), and carbapenemases, and has numerous efflux pumps, all of which synergistically make this bacterium extremely drug-resistant [18,19]. Its ability to form strong biofilms with a robust quorum-sensing system has enabled this bacterium to resist disinfectants and antimicrobials and successfully persist in the host environment to establish chronic infections [2,18,20]. Certain lineages of *P. aeruginosa,* such as ST235 and ST175, have emerged as main agents of serious nosocomial infections [21,22].

## 2. Antimicrobial Resistance Mechanisms

Antimicrobial agents are purportedly meant to eliminate microorganisms, particularly those deleterious to the health of plants, animals, and humans. Besides these, antimicrobials are also useful in preserving foods from spoilage. Antimicrobial agents could be of chemical or natural origin. The antibiotic penicillin is recognized as the first microbe-derived antimicrobial compound employed clinically to treat infectious diseases. The three-decade period following the discovery of penicillin is termed the “golden period of antimicrobial therapy”, as numerous antibiotics were discovered and inducted into clinical use [23]. However, along with the discovery of antimicrobials, bacteria that survived and grew in antibiotics were also discovered, suggesting that such bacteria can be present in the environment or possibly evolve during antibiotic exposure. Penicillin-resistant staphylococci were isolated from clinical samples in the early 1940s, and with the widespread use of antibiotics in the following two decades, the emergence and spread of antibiotic-resistant strains increased dramatically in clinical settings [24]. The development of resistance followed the discovery of new antimicrobials, and this phenomenon is often aided by the development of cross-resistance to closely related antibiotics or antibiotics belonging to the same group.

The bacterial mechanisms of antibiotic resistance are diverse and are broadly classified into (i) enzymatic inactivation of antibiotics, (ii) modification of drug target, (iii) deceased drug permeability, and (iv) active efflux of antibiotics [25]. The enzymatic inactivation is achieved by hydrolysis of antibiotics by bacterial enzymes such as the β-lactamases that degrade β-lactam antibiotics such as the penicillins, cephalosporins, and carbapenems, macrolide esterases, and the fosfomycin-inactivating enzymes. The second mechanism of enzymatic inactivation of antibiotics involves the structural modification of drugs by group transfer activities from enzymes such as acetyltransferases, phosphotransferases, glycosyltransferases, and nucleotidyltransferases [26,27]. The group transfer mechanism results in the irreversible modification of the antibiotic structure, which can no longer bind to its target. The antibiotics susceptible to this resistance mechanism include chloramphenicol, aminoglycosides, macrolides, and fosfomycin [23].

Alternatively, the antibiotic targets may be modified to circumvent binding by the antibiotics. This resistance may be achieved by mutations in the genes encoding the proteins which act as antibiotic targets, such as the penicillin-binding proteins (PBPs), the modification of which results in the inability of β-lactams to bind to PBPs resulting in resistance development [28]. Mutations in QRDRs (quinolone resistance determinant regions) result in the modification of ribosomal targets leading to the development of resistance to quinolone–fluoroquinolone antibiotics [29].

The outer membrane porins often regulate the entry of antimicrobials into the bacterial cell, the structural modification of which can result in reduced permeability of drugs through the cellular membrane. Such porin modifications result from mutations in porin-encoding genes under antibiotic pressure, and bacteria expressing defective porin proteins resist the entry of antibiotics such as the aminoglycosides, chloramphenicol, tetracyclines, β-lactams, and fluoroquinolones [30].

The efflux-mediated antimicrobial resistance mechanism involves the transmembrane proteins, which transport structurally diverse substrates across the membrane and primarily function to extrude toxic metabolites, Kreb’s cycle intermediates such as salts, sugars, vitamins, fatty acids, and amino acids, among others [9,31]. Based on the source of energy that drives this efflux process, the transporter proteins are broadly grouped into primary active transporters, which use ATP, and secondary active transporters, which utilize the concentration gradient established across the membrane by the primary active transport and respiration [32,33].

## 3. Antimicrobial Transporter Superfamilies

Many of the transport systems used by microorganisms can fall into one of a variety of protein superfamilies, the vast majority of which have been incorporated into a massive transporter classification database (TCDB) [34]. These and other biological transport systems have been systematically and taxonomically organized and are readily accessible in the TCDB, a continually updated resource [35]. The transporter superfamilies are established according to similarities in evolutionary origin, sequence, protein structure, and energization modes [36,37].

**Figure 1:**
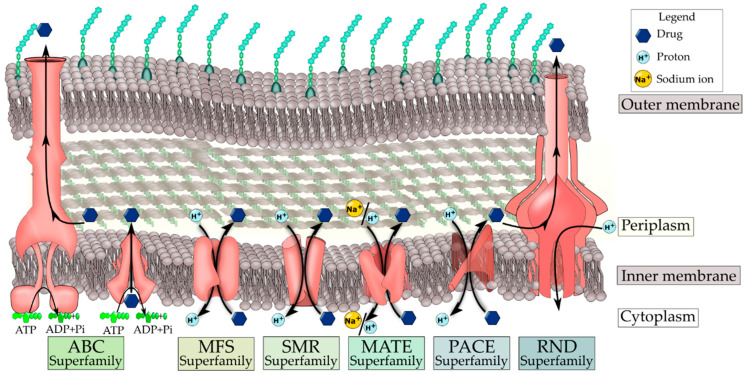
Bacterial antimicrobial transporter superfamilies. The ABC transporter superfamily consists of primary active transport systems that use ATP hydrolysis to drive antimicrobial efflux from bacterial cells. The antimicrobial efflux pumps belonging to the MFS, SMR, MATE, and PACE use ion/drug antiport mechanisms to extrude antimicrobial agents from the cytoplasm. The RND superfamily transporters are multi-component systems to efflux antimicrobial solutes to the external milieu of the bacterium.

Solute transporters that involve antimicrobial agents and bacterial pathogens can be grouped into a handful of superfamilies. The drug/metabolite transporter (DMT) superfamily consists of many protein families, such as the small multidrug resistance (SMR) family [38]. The multidrug/oligosaccharidyl-lipid/polysaccharide (MOP) superfamily harbors [39] several distantly related protein families, one of which is the multidrug and toxic compound extrusion transporters (MATE) family consisting of numerous cation-based multidrug antiporters [40]. The resistance–nodulation–cell division (RND) superfamily contains many solute transporters, many of which are found in bacterial pathogens conferring resistance to multiple antimicrobial agents [41]. The proteobacterial antimicrobial compound efflux (PACE) family encompasses members that are multidrug efflux pump systems in bacteria [42]. One of the largest groups is the major facilitator superfamily (MFS), consisting of secondary active transporters and passive facilitators [43]. This review focuses primarily on the multidrug efflux pumps of the MFS as they are extensively studied, ubiquitous across all living taxa, and involved in conferring drug resistance in clinical pathogens, making them good targets for modulation [9,44]. The extensively studied bacterial antimicrobial transporter superfamilies are illustrated in Figure 1.

### 3.1. Major Facilitator Superfamily (MFS)

**Figure 2:**
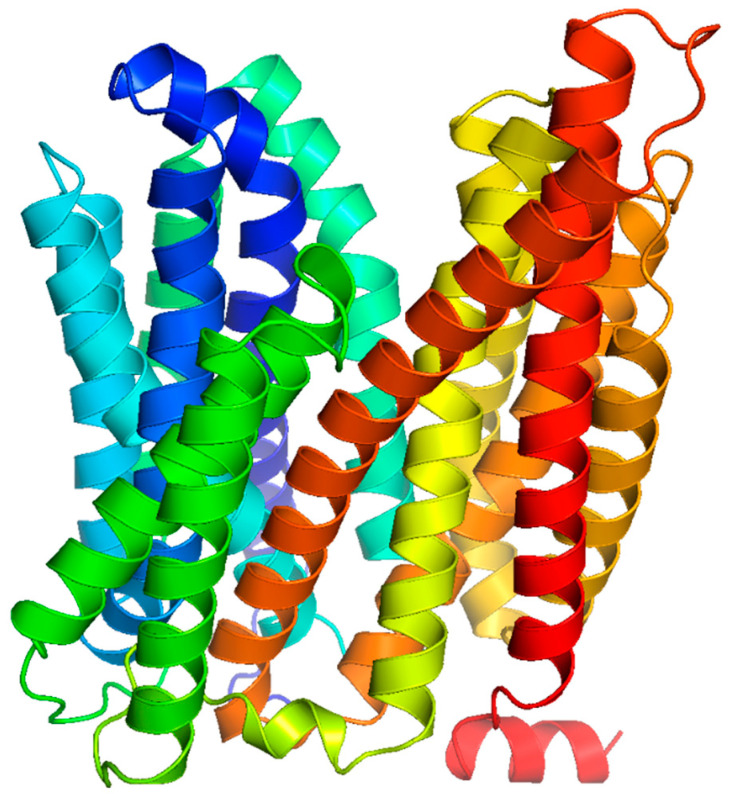
Predicted structure of LmrS from *S. aureus*. The LmrS multidrug efflux pump of the MFS has 14 predicted transmembrane domains that are α-helical in nature.

The MFS solute transporters consist of passive and secondary active transporters such as uniporters, symporters, and antiporters [45]. The protein members of the MFS typically have between 300 and 600 amino acids with 12 or 14 transmembrane domains composed of α-helices [46]. For instance, the LmrS multidrug efflux pump from *S. aureus* is predicted to harbor 14 membrane-spanning helices, with the N- and C-termini facing the cytoplasmic side of the cell membrane, Figure 2.

The MFS transporters share similarities in amino acid sequences [47], including several functional, highly conserved sequence motifs [48] and protein structures. The MFS transporters have diverse, structurally distinct membrane transport substrates, such as sugars, amino acids, metabolic intermediates, nucleic acids, ions, and antimicrobial agents [47].

#### MFS Antimicrobial Efflux Pump Structure and Function

The first transporter of the MFS to be characterized physiologically and at the molecular level was the *E. coli* tetracycline efflux pump, TetA, discovered in the laboratory of Levy [49,50]. Shortly afterward, related tetracycline transporters were found in Gram-negative and -positive microorganisms, collectively called TetA, with sub-classes including A-D and others [51]. Interestingly, it was discovered that these so-called single-substrate TetA transporters were homologous to bacterial multidrug efflux pumps, such as NorA [52], MdfA [53], and QacA [54], which are among the now most intensively studied antimicrobial transporters of the MFS.

High-resolution protein structures have been elucidated for several multidrug efflux pumps of the MFS. Multidrug efflux pumps of the MFS that have been crystalized and structures determined include EmrD [55], YajR [56,57], MdfA [53,58], and SotB [59], all from *E. coli*, and more recently, NorC from *S. aureus* [60] and LmrP from *Lactococcus lactis* [61]. These MFS drug transport proteins generally have two global domains, the N-terminus and the C-terminus domains, each harboring six-helical bundles [62] related by a so-called twofold pseudosymmetrical axis that runs perpendicular to the membrane bilayer [63]. This structural motif, called the MFS fold, appears in many multidrug efflux pump proteins and symporters of the superfamily [64,65]. Furthermore, these antimicrobial transporters alternately expose their substrate binding sites on either side of the membrane to mediate drug and ion translocation across the membrane during the transport cycle, a catalytic mechanism known as the alternating-access model [66,67].

Thus far, the MFS antimicrobial and multidrug efflux pumps share a highly conserved signature sequence called motif C [68] and the antiporter motif [69,70] in the fifth transmembrane helix. The antiporter motif C has the consensus amino acid sequence of “G (X)_8_ G (X)_3_ G P (X)_2_ G G” [71] consisting largely of a highly hydrophobic region with a proline manifested as a so-called GP dipeptide, where X is any amino acid [69,70]. The residues of the antiporter motif appear to play major mechanistic roles during drug and multidrug transport [48]. The first evidence to demonstrate the functional importance of the antiporter motif was reported on TetA(C), in which the highly conserved residue Gly-147, part of the GP dipeptide, was necessary for tetracycline resistance [70]. Since then, other transporters have been reported to have a variety of functional roles attributable to residues of motif C, such as mediating the direction of substrate transport [72], change in protein conformation during transport [73], forming an ion or substrate leak barrier [74,75,76], protein stabilization [77], drug binding [78], forming an accessible central cavity for binding substrates [79], and constituting the interface boundary between the two helical bundles [80] of the MFS antimicrobial efflux pumps. More recently, it was reported that motif C’s residues form a flexible hinge structure that undergoes conformational changes during transport and a regulator switch mechanism for that hinge’s conformation change [81]. As new studies are needed to fully understand the mechanisms played by the antiporter motif C structure, it has become an important target for resistance modulation [44], especially in multidrug-resistant bacterial pathogens [82,83].

Another widely studied conserved sequence motif is referred to as motif A with the consensus sequence “G (X)_3_ D R/K X G R R”, which is present in the intracellular loop between helices two and three of the vast majority of transporters of the MFS [36,47,84]. These conserved residues are found in symporters of the MFS, reviewed elsewhere [48]. The laboratories of Yamaguchi and Levy were the first to independently report evidence for a functional role of motif A residues in an antimicrobial efflux pump of the MFS [85,86]. They showed that the dipeptide Ser-Asp in the loop conferred drug transport and formed a gate in the tetracycline pumps TetA(B) and TetA(C) [51,87]. Analyses of related MFS multidrug efflux pumps from a structure–function approach in many laboratories have provided physiological evidence for residues of motif A. For example, the structures formed by the motif A consensus sequence were shown to confer the substrate pathway through the transporters [88,89], structural stability mediated by salt bridges [56], conformation change regulation [88,89], an interface boundary between the N- and C-terminal bundles [56], an electrochemical ion-gradient potential sensor [90,91], and a switch that influences binding site orientation [91]. These studies further implicate a critical solute transport role in virtually all protein members of the MFS. Continued analyses of the residues are anticipated to shed new insight into the molecular mechanism of drug transport across the membrane and the generation of mutants with altered specificities for transporting desirable substrates. Thus, the residues of motif A and the structure and functions conferred by them continue to be a focus in mechanistic studies of solute transport in proteins of the MFS [23,92,93].

## 4. Efflux Pump Inhibition

Multidrug efflux pumps of the MFS are known to confound treatment efforts toward clinical bacterial infections [23]. Thus, understanding the modulation of antimicrobial agent efflux by MFS drug pumps in potentially pathogenic microorganisms is of considerable interest to restore the clinical efficacy of treatment of infections caused by multidrug-resistant pathogens [44,94]. Naturally occurring plant-derived agents as resistance modulators in such cases represent a promising avenue [31]. Our laboratory discovered that a plant-derived extract from *Allium sativum* and its bioactive agent, allyl sulfide, reduced resistance to substrates and drug transport activity of the MFS multidrug efflux pump EmrD-3 from *Vibrio cholerae* [95]. In the same study, we demonstrated that *A. sativum* extract showed a synergistic reduction in the antimicrobial susceptibility in host cells harboring EmrD-3, pointing to this resistance mechanism as a suitable target for the modulation of resistance in severe cholera infections [96]. In another study, we found extracts from the common food spice *Cuminum cyminum* directly inhibited the transport activity of the LmrS multidrug efflux pump from *S. aureus*. We showed that the compound cumin aldehyde restored the susceptibility levels of antimicrobial agents that are known substrates of the LmrS multidrug efflux pump [97]. Therefore, the MFS transporter LmrS is another desirable target for developing putative modulatory agents against infection from MRSA [1,9,94].

The NorA multidrug efflux pump system of *S. aureus* has been a good target for transport inhibition studies reviewed elsewhere [98]. In *S. aureus*, plant-derived chalcones showed synergistic activity with antimicrobial agents ciprofloxacin, norfloxacin, and ethidium bromide involving the MFS proteins NorA and MepA [99]. Molecular docking analysis of these chalcones showed close interactions with NorA residues Ser-337, Met-338, Gly-339, and Asn-340, implicating these residues as good molecular targets for transport studies and resistance modulation [99]. The affected residues directly involve ciprofloxacin and norfloxacin binding and transport across the membrane through NorA. Recently, it was reported that the saponin secondary metabolite hecogenin acetate, while demonstrating antibacterial effects on isolates of *S. aureus*, nevertheless failed to show an inhibitory effect on drug efflux activities in NorA and MepA [100].

Interestingly, evaluation of the 1,8-naphthyridine sulfonamides showed that they could inhibit both β-lactamases and the QacC and QacA/B efflux pumps in *S. aureus* [101]. Thus, these agents and their derivatives show promise in addressing infection by pathogens by targeting more than one resistance mechanism, as in the case of resistances conferred by enzymatic inactivation and active drug efflux. More recently, berberine, a naturally occurring plant compound and known efflux pump inhibitor of Mdr1p, an MFS multidrug efflux pump from *Candida albicans* [102], reduced the resistance to multiple antimicrobial agents conferred by MdfA of *E. coli* [103]. Using a combination of molecular simulation dynamics and physiological analysis of transport by MdfA across the membrane, berberine was reported to affect the formation of salt bridges and alter the hydrophobic interactions of MdfA with water in the membrane during the transport cycle to inhibit transport [103]. Another study showed that resistance of *C. albicans* to the antifungal agent fluconazole conferred by the MFS pump Mdr1 could be blocked by plant extracts from *Acalypha communis* and *Solanum atriplicifolium.* Extracts from Argentinian native plants reverse fluconazole resistance in *Candida* species [104]. Although the biochemical nature of these plant extracts was not identified in the study, the inhibitory effects on Mdr1 are of interest, as these extracts are considered non-toxic and potent in their activities. Table 1 shows the potential efflux pump inhibitors that hold tremendous promise in restoring the antimicrobial susceptibility of important bacterial pathogens.

## 5. Antimicrobial Resistance and Biofilms

### Dynamics between Biofilm Formation and Antibiotic Resistance

Bacteria form biofilms in most scenarios where the dispersal and planktonic stages are considered intermediate or transitional. Biofilms are reported from the sea, rivers, food processing surfaces, medical implants, and the International Space Station [141]. Biofilms are medically important since almost 65–80% of human chronic infections are attributed to pathogenic biofilms [142]. The biofilm has become a menace in the food processing industry due to its persistence on food contact surfaces [143]. Biofilms are a survival strategy for bacteria to escape environmental stress, including predation by the bacteriophage [144]. Environmental stress triggers the transition of free-swimming planktonic forms to sessile forms that attach to a biotic or abiotic surface [145].

There are various aspects in the relationship between antibiotic resistance and biofilm formation. One aspect is that the biofilm acts as the antibiotic resistance gene pool, thus facilitating the emergence of antibiotic resistance bacteria. Another is that the biofilms enhance the survivability of drug-resistant bacteria in a harsh environment, thus helping them to sustain in various environments for a long time. A study conducted in the Yangtze Estuary found that antibiotic-resistant genes (ARG) were high in biofilms, followed by sediment and water. The biofilm is an evident sink for ARG [146]. Hence, biofilm acts as a reservoir of antibiotic-resistant genes, thus facilitating antibiotic resistance in the bacterial biofilm community. Ratajczak et al. [147] found a positive correlation between MDR and biofilm formation in *P. aeruginosa*. Another aspect is that biofilms are a survival strategy for bacteria with low levels of antimicrobial resistance.

A statistically negative correlation was observed between biofilm formation and MDR in *Acinetobacter baumanii*. The MDR and XDR isolates formed weak biofilms compared to non-MDR isolates producing robust biofilms [148]. Exposure to sub-inhibitory and sub-lethal concentrations of different antimicrobials triggers biofilm formation in different bacteria. A study conducted with *P. aeruginosa* found that exposure to aminoglycosides, particularly tobramycin, had the most effect on biofilm formation. Neither polymyxin B, a peptide antibiotic cationic like the aminoglycosides, nor carbenicillin or chloramphenicol had any effect on biofilm formation. Thus, *P. aeruginosa* forms biofilm as a specific response to the aminoglycoside antibiotics [149]. A recent study shed a different light on understanding antimicrobial resistance in biofilms. The zone of inhibition formed during tobramycin disc diffusion resulted from the transition of *P. aeruginosa* from planktonic to biofilm growth mode [150]. The bacterial biofilm confers protection from antibiotics through various means. Antimicrobial resistance or tolerance can be grouped into extracellular, cellular, and nuclear components, i.e., the biofilm matrix, the physiological state of bacteria, and genetic determinants. Resistant microorganisms can grow in the presence of a bactericidal or bacteriostatic antimicrobial agent at a concentration normally inhibitory to growth measured as minimum inhibitory concentration (MIC).

**Figure 3:**
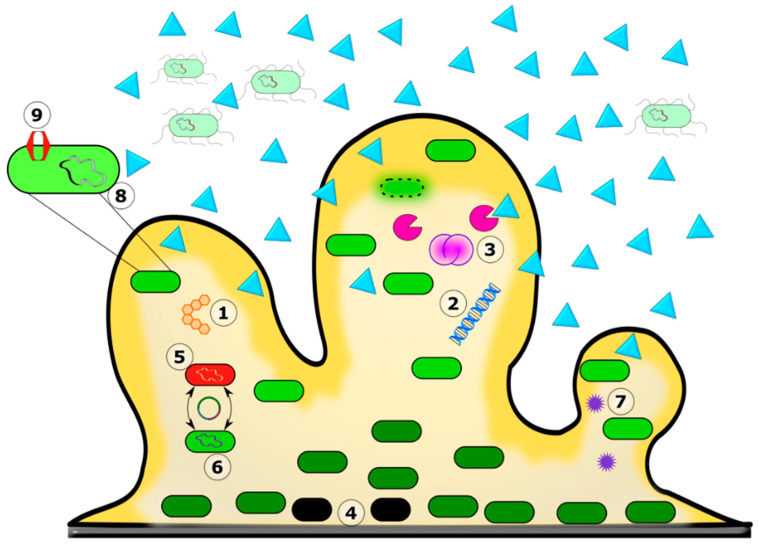
Mechanism of antibiotic recalcitrance in the biofilm matrix. The biofilm’s color gradient (yellow) depicts the availability of nutrients and oxygen from high to low and darker to lighter shades. The color differentiation in the bacterial cells depicts their physiological state, with the lighter ones in active phases and the darker green ones in less active stationary phases. Biofilm greatly reduced the diffusion of certain antibiotic molecules (blue triangles). 1. The exopolysaccharide. 2. eDNA. 3. Bacterial autolysis releases antibiotic-binding and degrading molecules into the matrix. 4. Persister cells. 5. Mixed species in the biofilm. 6. Horizontal gene transfer and increased frequency of mutation. 7. Quorum sensing. 8. Biofilm-specific antibiotic resistance genes. 9. Efflux pump.

In contrast, tolerance to an antimicrobial agent is the ability of a microorganism to survive but neither grow nor die in the presence of a bactericidal antimicrobial agent measured as minimum bactericidal concentration (MBC) [151]. The reduced susceptibility of biofilm to antibiotics could be due to complex interactions, and hence a clear demarcation as antibiotic resistance or tolerance might not be feasible in all the settings. Hence many authors use the term recalcitrance to denote the reduced susceptibility of the biofilm community to the antibiotics tested [152,153,154]. The mechanisms involved in antibiotic recalcitrance in a biofilm are depicted in Figure 3.

## 6. Role of the Biofilm Matrix in Antibiotic Recalcitrance

The biofilm matrix comprises components such as the extracellular polymeric substance (EPS), extracellular DNA (eDNA), proteins, and lipids inside which the bacterial cells are embedded. In most biofilms, the bacterial cells account for less than 10% of the biofilm’s dry mass [155]. The biofilm composition differs between species and even within the species. The MRSA biofilms are more proteinaceous compared to the polysaccharide-rich MSSA biofilms [156].

## 7. MFS Transporters and Biofilms

Efflux pumps play important roles in the formation of biofilms, as well as in the antibiotic resistance of biofilm bacteria. The overexpression of tetracycline resistance efflux pump TetA(C) in *Escherichia coli* biofilm contributes to forming mature biofilms, stress tolerance, and antimicrobial resistance [157]. The inactivation of efflux pumps abolishes biofilm formation, suggesting that efflux pumps as essential for biofilm formation and its persistence [158]. Pinostrobin, a plant-derived flavonoid compound, has anti-efflux and anti-biofilm activities. This compound supposedly interacts more efficiently with MFS efflux pumps of Gram-positive bacteria, reducing the MIC of ciprofloxacin by 128 times in MRSA [159]. In *Salmonella enterica* serovar Typhimurium, efflux pump knockout mutants lacking EmrAB or MdfA were found to be deficient in biofilm formation due to the mutants’ inability to produce curli, an essential component of biofilm matrix [160]. Boeravinone B, a known NorA multidrug efflux pump inhibitor, also inhibited biofilm formation by *S. aureus* [134].

Since biofilm formation is strongly associated with the quorum-sensing mechanism of bacteria, efflux pumps promote biofilm formation by reducing the impact of antibacterials and extrusion of quorum-sensing signaling molecules [161,162]. Efflux pump genes are overexpressed in biofilms, and this corresponds with the overexpression of quorum-sensing genes, suggesting a strong relationship between these two processes [163,164,165]. Biologically derived polyamines such as cadaverine, putrescine, spermidine, and spermine are important in bacteria for oxidative stress tolerance, biofilm formation, and persistence. These molecules are also substrates for efflux pumps, such as the AmvA protein of *Acinetobacter baumannii*, suggesting that the biofilm formation and efflux pump activities can be interrelated [166].

The efflux-pump-mediated biofilm formation is a complex process involving an interplay between multiple pathways, and the net effect of these interactions might vary in different bacterial species. For example, the inactivation of LmrB in *Streptococcus mutans* resulted in increased EPS secretion and biofilm formation while upregulating other efflux genes’ expression [167]. In many Gram-negative and -positive bacteria, efflux pumps contribute positively to biofilm formation [168]. Efflux pump inhibitors (EPI), in many instances, not only enhance the susceptibility of bacteria to antimicrobials but also reduce or inhibit biofilm formation [169]. Efflux pumps purportedly contribute to biofilm formation at various stages, such as (i) the initiation of biofilm formation in which quorum-sensing molecules play important roles, and the overexpressed efflux pumps might participate in extruding quorum-sensing molecules to bring about a desired effect; (ii) biofilm maturation, during which toxic metabolites need to be expelled from the cellular environment; and (iii) biofilm persistence, in which the biofilm bacteria are protected from an antimicrobial and noxious substance in the surrounding environment [168,170].

The quorum-sensing system also regulates the expression of virulence genes, suggesting that antibiotic resistance, biofilm formation, virulence, and persistence of pathogenic bacteria stay interlinked and provide opportunities to identify the means of interfering with these processes with the ultimate goal of pathogen control [171]. Apart from clinical implications, efflux pump inhibition and the consequent quorum-sensing inhibition could positively impact the shelf life of highly perishable products such as fish. A recent report suggests that black pepper essential oil (BPEO) and its bioactive compounds, limonene (LIM) and β-caryophyllene (CAR), could inhibit efflux pumps and quorum sensing in the fish spoilage bacterium *Pseudomonas psychrophila* and reduce its spoilage potential [172]. Compounds such as thioridazine and chlorpromazine significantly reduced the gene expression of efflux pumps such as *norB*, *norC*, *abcA*, and *mepA* and impaired their ability to form biofilms [173]. Copper nanoparticles (CuNPs) have also been shown to inhibit efflux pumps and biofilm formation in *S. aureus* and *P. aeruginosa* [174].

Similarly, toluidine blue O (TBO)-mediated photodynamic therapy (PDT) resulted in decreased expression of the *norA, norB, sepA, mepA,* and *mdeA* efflux pump genes and impaired biofilm formation by *S. aureus* strains [175]. Menadione (vitamin K3) has been recently shown to have EPI activity on *norA* involving two pathways: direct interaction with the NorA protein and indirectly affecting the expression of the *norA* gene [135]. Nilotinib, a tyrosine kinase inhibitor, significantly reduced *S. aureus* biofilm formation when combined with ciprofloxacin, suggesting that this compound interacted with the NorA efflux pump leading to diminished activity [176]. The antifungal ketoconazole is an inhibitor of the NorA efflux pump and biofilm formation in *S. aureus* [177]. MFS efflux pumps are important for biofilm formation in *E. coli* as evidenced by deficient biofilm formation by the mutant *E. coli* K12 strain lacking *emrD, emrE, emrK, acrD, acrE,* and *mdtE* efflux pump genes [178]. In *Shigella flexneri*, the efflux pump EmrKY contributes to intracellular survival in macrophages, and its loss results in reduced biofilm formation and increased susceptibility to DNA-damaging substances [179].

## 8. Future Directions

One new field of study involves the modulation of bacterial antimicrobial efflux pump alteration in expression by nanoparticles [180,181]. We anticipate that this area shows further promise toward generating new treatment strategies against potentially untreatable infections caused by multidrug-resistant pathogens [182]. Surprisingly, although many studies show inhibition of multidrug resistance in transporters of the MFS [9,23,92,93,183], few, if any, of these modulatory agents have reached clinical trials, suggesting that a focus on this area of infectious disease investigation is lacking. The reasons for this apparent disparity are unclear.

One promising avenue can be found in the continued analysis of the MFS antimicrobial transporters’ conserved amino acid signature sequences, especially those transporters expressed in pathogenic microorganisms. Conserved amino acids of the MFS solute transporters represent functionally important aspects that drive multidrug efflux. Studying these drug efflux pumps’ structure–functional natures can reveal critical physiological systems conferring antimicrobial resistance. These molecular mechanisms of antimicrobial transport across the membrane are good targets for developing efflux pump inhibitors [184].

## Figures and Tables

**Table 1:** Natural compounds as efflux pump inhibitors (EPIs) against efflux pumps of the MFS family.

Inhibitor	Efflux Pump	Reference
Plant-derived alkaloid compounds (reserpine, piperines, and piperine analogs)	NorA, Bmr, MdeA, LmrA, PmrA	[105,106,107]
Flavonoids (genistein, sarothrin)	NorA	[108,109]
Flavones (chrysosplenol-D and chrysoplenetin)	NorA	[110]
Diterpenes (ferruginol)	NorA	[111,112]
Isolflavones	NorA	[113]
Coumarins	NorA	[114]
Kaempferol rhamnoside	NorA	[115]
2,6-dimethyl-4-phenyl-pyridine-3,5-dicarboxylic acid diethyl ester	NorA, MsrA	[116]
Indirubin	NorA	[117]
Chalcones (4-phenoxy-4′-dimethylamino ethoxy chalcone)	NorA	[118,119]
Oligosaccharides (orizabin)	NorA	[120]
Derivatives of 2-phenylquinoline	NorA	[121,122,123]
Abietane diterpenes	Tet(K), Msr(A)	[124]
Essential oils	TetK	[125]
5′-Methoxyhydnocarpin-D and Pheophorbide A	NorA	[126]
Cumin seed oil, cumin aldehyde	LmrS	[127]
Plant-derived alkaloid compounds (berberine and palmatine)	NorA, MdfA	[103,128,129]
Sarothrin	NorA	[108]
Olympicin	NorA	[130]
Murucoidins	NorA	[131]
Clerodane diterpene 16α- hydroxycleroda-3,13 (14)-Z-dien-15,16-olid 6	NorB, NorC	[132]
Verapamil, capsaicin, boeravinone B	NorA, QacA	[133,134]
Cholecalciferol and alpha-tocopherol	TetK, MsrA	[101,135]
Carnosic acid	MsrA, TetK, and NorA	[136,137]
Linoleic and oleic acids	MsrA	[138]
Epigallocatechin gallate, Epicatechin gallate	TetK	[139]
Osthtol	NorA, MdeA, TetK, MsrA	[140]
